# *catena*-Poly[[[diaqua­sodium]-di-μ-aqua] 2-(2-pyrid­yl)quinoline-4-carboxyl­ate]

**DOI:** 10.1107/S1600536809053999

**Published:** 2010-01-09

**Authors:** Hu-Jun Hao, Cui-Wu Lin, Xian-Hong Yin, Fei-Long Hu, Yue Zhuang

**Affiliations:** a College of Chemistry and Ecological Engineering, Guangxi University for Nationalities, Nanning 530006, People’s Republic of China; b School of Chemistry & Chemical Engineering, Guangxi University, Nanning 530004, People’s Republic of China

## Abstract

In the title compound, [Na(H_2_O)_4_](C_15_H_9_N_2_O_2_), the Na^+^ ion is coordinated by six water mol­ecules in an octa­hedral geometry. The NaO_6_ octa­hedra are connected by sharing edges, forming a cationic chain along the *b*-axis direction. O—H⋯O and O—H⋯N hydrogen bonds link the chains and the 2-(2-pyrid­yl)quinoline-4-carboxyl­ate anions into a two-dimensional network parallel to (100).

## Related literature

For the syntheses of sodium 2-(2-pyrid­yl)quinoline-4-carboxyl­ate and 2-(2-pyrid­yl)quinoline-4-carboxylic acid, see: Bass *et al.* (1997 ▶); Convers *et al.* (2004 ▶). For the structures of 2-(2-pyrid­yl)-4-methyl­carboxy­quinoline and its Ru complex, see: Farah *et al.* (2003 ▶).
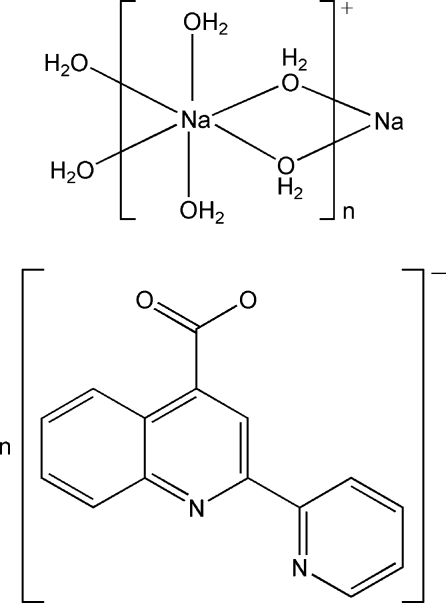


## Experimental

### 

#### Crystal data

[Na(H_2_O)_4_](C_15_H_9_N_2_O_2_)
*M* *_r_* = 344.30
Monoclinic, 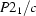

*a* = 19.0409 (17) Å
*b* = 5.2987 (5) Å
*c* = 16.8305 (16) Å
β = 103.107 (5)°
*V* = 1653.8 (3) Å^3^
*Z* = 4
Mo *K*α radiation
μ = 0.13 mm^−1^
*T* = 296 K
0.43 × 0.35 × 0.30 mm

#### Data collection

Siemens SMART 1000 CCD diffractometer
Absorption correction: multi-scan (*SADABS*; Sheldrick, 1996 ▶) *T*_min_ = 0.944, *T*_max_ = 0.962
14472 measured reflections
3647 independent reflections
2902 reflections with *I* > 2σ(*I*)
*R*_int_ = 0.028

#### Refinement

*R*[*F* ^2^ > 2σ(*F* ^2^)] = 0.035
*wR*(*F* ^2^) = 0.103
*S* = 1.05
3647 reflections
218 parameters
H-atom parameters constrained
Δρ_max_ = 0.30 e Å^−3^
Δρ_min_ = −0.19 e Å^−3^

### 

Data collection: *SMART* (Siemens, 1996 ▶); cell refinement: *SAINT* (Siemens, 1996 ▶); data reduction: *SAINT*; program(s) used to solve structure: *SHELXS97* (Sheldrick, 2008 ▶); program(s) used to refine structure: *SHELXL97* (Sheldrick, 2008 ▶); molecular graphics: *DIAMOND* (Brandenburg, 1999 ▶); software used to prepare material for publication: *SHELXTL* (Sheldrick, 2008 ▶).

## Supplementary Material

Crystal structure: contains datablocks I, global. DOI: 10.1107/S1600536809053999/hy2255sup1.cif

Structure factors: contains datablocks I. DOI: 10.1107/S1600536809053999/hy2255Isup2.hkl

Additional supplementary materials:  crystallographic information; 3D view; checkCIF report

## Figures and Tables

**Table 1:** Hydrogen-bond geometry (Å, °)

*D*—H⋯*A*	*D*—H	H⋯*A*	*D*⋯*A*	*D*—H⋯*A*
O3—H50⋯N2	0.85	2.02	2.8657 (14)	172
O3—H51⋯O2^i^	0.85	1.92	2.7722 (14)	174
O4—H4*A*⋯O5^i^	0.85	1.98	2.8222 (14)	172
O4—H4*B*⋯O1^ii^	0.85	1.91	2.7494 (14)	171
O5—H5*A*⋯O2	0.82	2.04	2.8093 (14)	156
O5—H5*B*⋯O2^ii^	0.85	1.97	2.8243 (13)	178
O6—H6*A*⋯O1^iii^	0.85	2.10	2.8914 (14)	156
O6—H6*B*⋯O3^iv^	0.85	2.00	2.8321 (14)	168

Symmetry codes: (i) 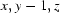; (ii) 
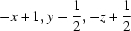
; (iii) 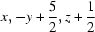; (iv) 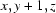.

**Fig. 1.**
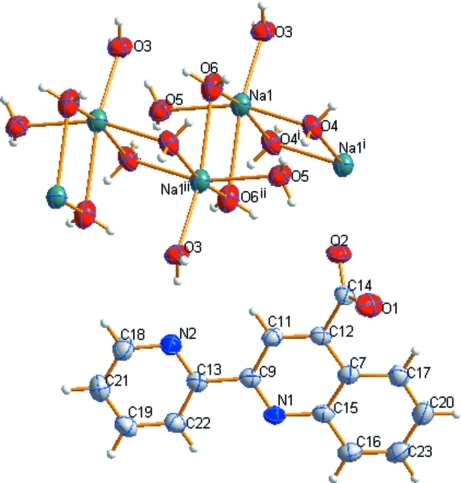
The structure of the title compound. Displacement ellipsoids are drawn at the 50% probability level. [Symmetry codes: (i) 1 - x, 1 - y, 1 - z; (ii) 1 - x, 2 - y, 1 - z.]

**Fig. 2.**
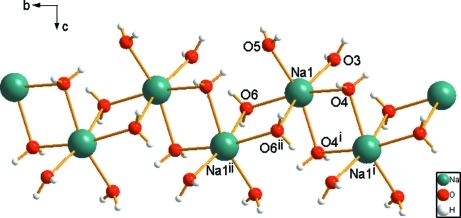
The chain of NaO6 octahedra along the b axis. [Symmetry codes: (i) 1 - x, 1 - y, 1 - z; (ii) 1 - x, 2 - y, 1 - z.]

**Fig. 3.**
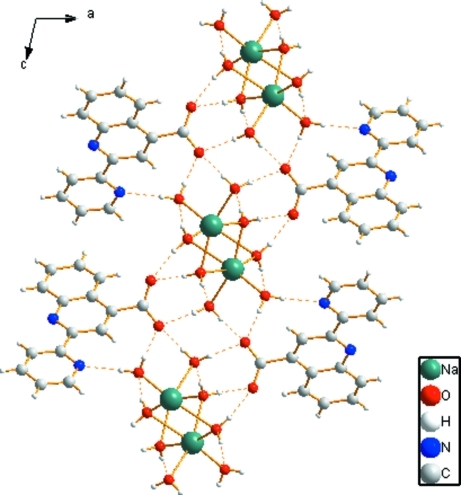
Crystal packing of the title compound viewed along the b axis. Dashed lines indicate hydrongen bonds.

## supplementary crystallographic information

### Comment

2-(2-pyridyl)quinoline-4-carboxylic acid is a 2,2'-bipyridyl-like ligand containing a carboxylate substituent, which represents a simple route to some important functionalities. The syntheses of sodium 2-(2-pyridyl)quinoline-4-carboxylate and 2-(2-pyridyl)quinoline-4-carboxylic acid have been reported (Bass *et al.*, 1997; Convers *et al.*, 2004). The structures of 2-(2-pyridyl)-4-methylcarboxyquinoline and its Ru complex have been reported by Farah *et al.* (2003). Here we present the structure of a sodium salt of 2-(2-pyridyl)quinoline-4-carboxylate in a tetrahydrate form.

The molecular structure of the title compound is shown in Fig. 1. The Na^I^ ion is coordinated by six water molecules in an octahedral geometry. Each coordination octahedron is connected with two adjacent ones by sharing edges, forming a cationic [Na(H_2_O)_4_]_n_ chain along the *b* direction (Fig. 2).

In the crystal structure, the cationic chains and the organic anions are linked through O–H···O and O–H···N hydrogen bonds into a layer structure (Table 1 and Fig. 3).

### Experimental

Sodium 2-(2-pyridyl)quinoline-4-carboxylate was prepared by a literature method (Bass *et al.*,1997). Colourless crystals were obtained by slow evaporation of an aqueous solution of this compound at room temperature.

### Refinement

H atoms bonded C atoms were positioned geometrically and refined as riding atoms, with C—H = 0.93 Å and with *U*_iso_(H) = 1.2*U*_eq_(C). H atoms of water molecules were found from difference Fourier maps and refined as riding atoms, with O—H = 0.85 Å and with *U*_iso_(H) = 1.5*U*_eq_(O).

### Crystal data

**Table:**

[Na(H_2_O)_4_](C_15_H_9_N_2_O_2_)	*F*(000) = 720
*M**_r_* = 344.30	*D*_x_ = 1.383 Mg m^−^^3^
Monoclinic, *P*2_1_/*c*	Mo *K*α radiation, λ = 0.71073 Å
Hall symbol: -P 2ybc	Cell parameters from 1202 reflections
*a* = 19.0409 (17) Å	θ = 2.2–27.2°
*b* = 5.2987 (5) Å	µ = 0.13 mm^−^^1^
*c* = 16.8305 (16) Å	*T* = 296 K
β = 103.107 (5)°	Block, colourless
*V* = 1653.8 (3) Å^3^	0.43 × 0.35 × 0.30 mm
*Z* = 4	

### Data collection

**Table:**

Siemens SMART 1000 CCD diffractometer	3647 independent reflections
Radiation source: fine-focus sealed tube	2902 reflections with *I* > 2σ(*I*)
graphite	*R*_int_ = 0.028
φ and ω scans	θ_max_ = 27.2°, θ_min_ = 2.2°
Absorption correction: multi-scan (*SADABS*; Sheldrick, 1996)	*h* = −24→24
*T*_min_ = 0.944, *T*_max_ = 0.962	*k* = −6→6
14472 measured reflections	*l* = −21→21

### Refinement

**Table:**

Refinement on *F*^2^	Primary atom site location: structure-invariant direct methods
Least-squares matrix: full	Secondary atom site location: difference Fourier map
*R*[*F*^2^ > 2σ(*F*^2^)] = 0.035	Hydrogen site location: inferred from neighbouring sites
*wR*(*F*^2^) = 0.103	H-atom parameters constrained
*S* = 1.05	*w* = 1/[σ^2^(*F*_o_^2^) + (0.0509*P*)^2^ + 0.3055*P*] where *P* = (*F*_o_^2^ + 2*F*_c_^2^)/3
3647 reflections	(Δ/σ)_max_ = 0.001
218 parameters	Δρ_max_ = 0.30 e Å^−^^3^
0 restraints	Δρ_min_ = −0.19 e Å^−^^3^

### Fractional atomic coordinates and isotropic or equivalent isotropic displacement parameters (Å^2^)

**Table:**

	*x*	*y*	*z*	*U*_iso_*/*U*_eq_	
Na1	0.46104 (3)	0.74129 (10)	0.43759 (3)	0.03736 (16)	
O4	0.53878 (5)	0.39638 (18)	0.42708 (6)	0.0400 (2)	
H4A	0.5271	0.2741	0.3940	0.060*	
H4B	0.5767	0.4622	0.4175	0.060*	
N1	0.12262 (5)	1.0815 (2)	0.20495 (6)	0.0327 (3)	
N2	0.21437 (6)	0.6022 (2)	0.34054 (7)	0.0374 (3)	
O6	0.40941 (6)	1.10971 (19)	0.47560 (6)	0.0480 (3)	
H6A	0.3835	1.1346	0.5099	0.072*	
H6B	0.3901	1.2170	0.4397	0.072*	
O5	0.49772 (5)	0.96277 (19)	0.33032 (6)	0.0414 (2)	
H5A	0.4620	1.0010	0.2950	0.062*	
H5B	0.5315	0.8953	0.3123	0.062*	
O3	0.36524 (5)	0.5014 (2)	0.36062 (6)	0.0427 (3)	
H50	0.3215	0.5470	0.3541	0.064*	
H51	0.3705	0.4282	0.3174	0.064*	
C7	0.20528 (7)	1.3150 (2)	0.14024 (7)	0.0304 (3)	
O2	0.38821 (5)	1.2386 (2)	0.22633 (6)	0.0442 (3)	
C9	0.17739 (6)	0.9467 (2)	0.24487 (7)	0.0301 (3)	
O1	0.34259 (5)	1.1583 (2)	0.09542 (6)	0.0528 (3)	
C11	0.24896 (7)	0.9854 (3)	0.23664 (8)	0.0326 (3)	
H11	0.2865	0.8904	0.2675	0.039*	
C12	0.26262 (6)	1.1624 (3)	0.18334 (7)	0.0305 (3)	
C13	0.15995 (7)	0.7438 (2)	0.29892 (7)	0.0314 (3)	
C14	0.33714 (7)	1.1890 (3)	0.16692 (8)	0.0341 (3)	
C15	0.13590 (7)	1.2676 (2)	0.15413 (8)	0.0314 (3)	
C16	0.07768 (7)	1.4199 (3)	0.11366 (9)	0.0402 (3)	
H16	0.0316	1.3888	0.1212	0.048*	
C17	0.21427 (8)	1.5153 (3)	0.08769 (8)	0.0389 (3)	
H17	0.2596	1.5482	0.0782	0.047*	
C18	0.19828 (8)	0.4159 (3)	0.38732 (9)	0.0439 (3)	
H18	0.2360	0.3186	0.4167	0.053*	
C19	0.07401 (8)	0.5048 (3)	0.35213 (9)	0.0415 (3)	
H19	0.0268	0.4718	0.3554	0.050*	
C20	0.15707 (8)	1.6597 (3)	0.05106 (9)	0.0449 (4)	
H20	0.1637	1.7919	0.0172	0.054*	
C21	0.12967 (8)	0.3599 (3)	0.39460 (9)	0.0426 (3)	
H21	0.1212	0.2273	0.4274	0.051*	
C22	0.08920 (7)	0.7007 (3)	0.30439 (8)	0.0387 (3)	
H22	0.0522	0.8031	0.2761	0.046*	
C23	0.08833 (8)	1.6110 (3)	0.06398 (9)	0.0446 (4)	
H23	0.0496	1.7104	0.0383	0.054*	

### Atomic displacement parameters (Å^2^)

**Table:**

	*U*^11^	*U*^22^	*U*^33^	*U*^12^	*U*^13^	*U*^23^
Na1	0.0418 (3)	0.0330 (3)	0.0387 (3)	0.0028 (2)	0.0121 (2)	0.0002 (2)
O4	0.0428 (6)	0.0361 (6)	0.0460 (6)	−0.0033 (4)	0.0199 (4)	−0.0019 (4)
N1	0.0270 (5)	0.0375 (7)	0.0341 (6)	0.0005 (5)	0.0081 (4)	−0.0017 (5)
N2	0.0330 (6)	0.0410 (7)	0.0399 (6)	0.0035 (5)	0.0116 (5)	0.0036 (5)
O6	0.0596 (7)	0.0408 (6)	0.0493 (6)	0.0129 (5)	0.0244 (5)	0.0061 (5)
O5	0.0392 (5)	0.0473 (6)	0.0385 (5)	0.0090 (5)	0.0105 (4)	0.0020 (4)
O3	0.0328 (5)	0.0516 (6)	0.0431 (5)	0.0054 (4)	0.0073 (4)	−0.0010 (5)
C7	0.0299 (6)	0.0330 (7)	0.0283 (6)	−0.0004 (5)	0.0065 (5)	−0.0040 (5)
O2	0.0270 (5)	0.0637 (7)	0.0421 (5)	−0.0008 (4)	0.0082 (4)	0.0001 (5)
C9	0.0277 (6)	0.0346 (7)	0.0286 (6)	0.0009 (5)	0.0081 (5)	−0.0035 (5)
O1	0.0405 (6)	0.0841 (8)	0.0389 (5)	0.0043 (5)	0.0197 (4)	−0.0019 (5)
C11	0.0270 (6)	0.0392 (8)	0.0321 (6)	0.0048 (5)	0.0075 (5)	0.0020 (5)
C12	0.0266 (6)	0.0372 (7)	0.0285 (6)	−0.0002 (5)	0.0078 (5)	−0.0042 (5)
C13	0.0300 (6)	0.0352 (7)	0.0301 (6)	0.0004 (5)	0.0092 (5)	−0.0036 (5)
C14	0.0297 (7)	0.0376 (8)	0.0373 (7)	0.0033 (5)	0.0126 (5)	0.0033 (5)
C15	0.0281 (6)	0.0340 (7)	0.0313 (6)	0.0001 (5)	0.0054 (5)	−0.0053 (5)
C16	0.0304 (7)	0.0451 (9)	0.0428 (8)	0.0047 (6)	0.0035 (6)	0.0002 (6)
C17	0.0395 (7)	0.0412 (8)	0.0368 (7)	−0.0029 (6)	0.0101 (6)	0.0007 (6)
C18	0.0430 (8)	0.0426 (9)	0.0472 (8)	0.0074 (6)	0.0126 (6)	0.0087 (6)
C19	0.0361 (7)	0.0478 (9)	0.0432 (7)	−0.0082 (6)	0.0146 (6)	−0.0019 (6)
C20	0.0521 (9)	0.0400 (8)	0.0407 (8)	0.0014 (7)	0.0068 (6)	0.0063 (6)
C21	0.0513 (9)	0.0369 (8)	0.0430 (8)	−0.0032 (7)	0.0177 (6)	0.0025 (6)
C22	0.0297 (7)	0.0473 (9)	0.0399 (7)	0.0009 (6)	0.0100 (5)	0.0034 (6)
C23	0.0426 (8)	0.0423 (9)	0.0440 (8)	0.0096 (6)	−0.0008 (6)	0.0023 (6)

### Geometric parameters (Å, °)

**Table:**

Na1—O6	2.3393 (11)	O2—C14	1.2544 (16)
Na1—O3	2.3559 (11)	C9—C11	1.4157 (17)
Na1—O4	2.3834 (11)	C9—C13	1.4935 (18)
Na1—O5	2.3867 (11)	O1—C14	1.2419 (16)
Na1—O4^i^	2.3909 (11)	C11—C12	1.3632 (18)
Na1—O6^ii^	2.6848 (13)	C11—H11	0.9300
Na1—Na1^i^	3.4262 (11)	C12—C14	1.5124 (17)
Na1—Na1^ii^	3.5661 (11)	C13—C22	1.3897 (18)
O4—H4A	0.85	C15—C16	1.4148 (18)
O4—H4B	0.85	C16—C23	1.357 (2)
N1—C9	1.3159 (17)	C16—H16	0.9300
N1—C15	1.3660 (17)	C17—C20	1.359 (2)
N2—C18	1.3408 (18)	C17—H17	0.9300
N2—C13	1.3409 (17)	C18—C21	1.372 (2)
O6—H6A	0.85	C18—H18	0.9300
O6—H6B	0.85	C19—C21	1.371 (2)
O5—H5A	0.82	C19—C22	1.383 (2)
O5—H5B	0.85	C19—H19	0.9300
O3—H50	0.85	C20—C23	1.399 (2)
O3—H51	0.85	C20—H20	0.9300
C7—C15	1.4152 (17)	C21—H21	0.9300
C7—C17	1.4167 (19)	C22—H22	0.9300
C7—C12	1.4187 (18)	C23—H23	0.9300
			
O6—Na1—O3	106.24 (4)	H50—O3—H51	108.7
O6—Na1—O4	165.19 (4)	C15—C7—C17	119.05 (12)
O3—Na1—O4	87.81 (4)	C15—C7—C12	116.97 (11)
O6—Na1—O5	90.56 (4)	C17—C7—C12	123.93 (12)
O3—Na1—O5	99.92 (4)	N1—C9—C11	122.72 (12)
O4—Na1—O5	91.59 (4)	N1—C9—C13	116.22 (11)
O6—Na1—O4^i^	84.49 (4)	C11—C9—C13	121.04 (11)
O3—Na1—O4^i^	101.12 (4)	C12—C11—C9	119.71 (12)
O4—Na1—O4^i^	88.28 (4)	C12—C11—H11	120.1
O5—Na1—O4^i^	158.94 (4)	C9—C11—H11	120.1
O6—Na1—O6^ii^	89.83 (4)	C11—C12—C7	119.27 (11)
O3—Na1—O6^ii^	163.58 (4)	C11—C12—C14	120.75 (12)
O4—Na1—O6^ii^	75.91 (4)	C7—C12—C14	119.91 (11)
O5—Na1—O6^ii^	82.92 (4)	N2—C13—C22	121.29 (12)
O4^i^—Na1—O6^ii^	76.62 (4)	N2—C13—C9	117.95 (11)
O6—Na1—Na1^i^	127.27 (4)	C22—C13—C9	120.76 (12)
O3—Na1—Na1^i^	96.20 (3)	O1—C14—O2	125.45 (12)
O4—Na1—Na1^i^	44.23 (3)	O1—C14—C12	116.92 (12)
O5—Na1—Na1^i^	132.11 (4)	O2—C14—C12	117.63 (11)
O4^i^—Na1—Na1^i^	44.05 (3)	N1—C15—C16	118.50 (11)
O6^ii^—Na1—Na1^i^	70.68 (3)	N1—C15—C7	122.94 (12)
O6—Na1—Na1^ii^	48.84 (3)	C16—C15—C7	118.57 (12)
O3—Na1—Na1^ii^	154.91 (4)	C23—C16—C15	120.79 (13)
O4—Na1—Na1^ii^	116.80 (3)	C23—C16—H16	119.6
O5—Na1—Na1^ii^	85.04 (3)	C15—C16—H16	119.6
O4^i^—Na1—Na1^ii^	76.28 (3)	C20—C17—C7	120.47 (13)
O6^ii^—Na1—Na1^ii^	40.99 (2)	C20—C17—H17	119.8
Na1—O4—Na1^i^	91.72 (4)	C7—C17—H17	119.8
Na1—O4—H4A	123.9	N2—C18—C21	124.12 (14)
Na1^i^—O4—H4A	109.5	N2—C18—H18	117.9
Na1—O4—H4B	105.6	C21—C18—H18	117.9
Na1^i^—O4—H4B	119.2	C21—C19—C22	118.97 (13)
H4A—O4—H4B	107.2	C21—C19—H19	120.5
C9—N1—C15	118.28 (11)	C22—C19—H19	120.5
C18—N2—C13	117.78 (12)	C17—C20—C23	120.49 (14)
Na1—O6—Na1^ii^	90.17 (4)	C17—C20—H20	119.8
Na1—O6—H6A	131.3	C23—C20—H20	119.8
Na1^ii^—O6—H6A	100.8	C19—C21—C18	118.11 (14)
Na1—O6—H6B	120.3	C19—C21—H21	120.9
Na1^ii^—O6—H6B	112.7	C18—C21—H21	120.9
H6A—O6—H6B	98.9	C19—C22—C13	119.71 (13)
Na1—O5—H5A	109.5	C19—C22—H22	120.1
Na1—O5—H5B	116.0	C13—C22—H22	120.1
H5A—O5—H5B	114.6	C16—C23—C20	120.62 (13)
Na1—O3—H50	122.1	C16—C23—H23	119.7
Na1—O3—H51	119.3	C20—C23—H23	119.7
			
O6—Na1—O4—Na1^i^	60.71 (16)	N1—C9—C13—C22	1.18 (18)
O3—Na1—O4—Na1^i^	−101.20 (4)	C11—C9—C13—C22	−177.58 (12)
O5—Na1—O4—Na1^i^	158.93 (4)	C11—C12—C14—O1	120.27 (15)
O4^i^—Na1—O4—Na1^i^	0.0	C7—C12—C14—O1	−56.85 (18)
O6^ii^—Na1—O4—Na1^i^	76.64 (4)	C11—C12—C14—O2	−59.32 (18)
Na1^ii^—Na1—O4—Na1^i^	73.68 (4)	C7—C12—C14—O2	123.56 (14)
O3—Na1—O6—Na1^ii^	176.58 (4)	C9—N1—C15—C16	−177.42 (12)
O4—Na1—O6—Na1^ii^	15.43 (16)	C9—N1—C15—C7	2.51 (18)
O5—Na1—O6—Na1^ii^	−82.92 (4)	C17—C7—C15—N1	−178.81 (12)
O4^i^—Na1—O6—Na1^ii^	76.58 (3)	C12—C7—C15—N1	−1.38 (18)
O6^ii^—Na1—O6—Na1^ii^	0.0	C17—C7—C15—C16	1.13 (18)
Na1^i^—Na1—O6—Na1^ii^	65.29 (5)	C12—C7—C15—C16	178.56 (12)
C15—N1—C9—C11	−0.61 (19)	N1—C15—C16—C23	178.51 (12)
C15—N1—C9—C13	−179.35 (10)	C7—C15—C16—C23	−1.4 (2)
N1—C9—C11—C12	−2.4 (2)	C15—C7—C17—C20	−0.1 (2)
C13—C9—C11—C12	176.26 (12)	C12—C7—C17—C20	−177.32 (13)
C9—C11—C12—C7	3.49 (19)	C13—N2—C18—C21	0.6 (2)
C9—C11—C12—C14	−173.65 (12)	C7—C17—C20—C23	−0.7 (2)
C15—C7—C12—C11	−1.68 (18)	C22—C19—C21—C18	−0.3 (2)
C17—C7—C12—C11	175.61 (12)	N2—C18—C21—C19	−0.7 (2)
C15—C7—C12—C14	175.49 (11)	C21—C19—C22—C13	1.3 (2)
C17—C7—C12—C14	−7.22 (19)	N2—C13—C22—C19	−1.5 (2)
C18—N2—C13—C22	0.5 (2)	C9—C13—C22—C19	177.24 (12)
C18—N2—C13—C9	−178.27 (12)	C15—C16—C23—C20	0.7 (2)
N1—C9—C13—N2	179.93 (11)	C17—C20—C23—C16	0.4 (2)
C11—C9—C13—N2	1.16 (18)		

Symmetry codes: (i) −*x*+1, −*y*+1, −*z*+1; (ii) −*x*+1, −*y*+2, −*z*+1.

### Hydrogen-bond geometry (Å, °)

**Table:**

*D*—H···*A*	*D*—H	H···*A*	*D*···*A*	*D*—H···*A*
O3—H50···N2	0.85	2.02	2.8657 (14)	172
O3—H51···O2^iii^	0.85	1.92	2.7722 (14)	174
O4—H4A···O5^iii^	0.85	1.98	2.8222 (14)	172
O4—H4B···O1^iv^	0.85	1.91	2.7494 (14)	171
O5—H5A···O2	0.82	2.04	2.8093 (14)	156
O5—H5B···O2^iv^	0.85	1.97	2.8243 (13)	178
O6—H6A···O1^v^	0.85	2.10	2.8914 (14)	156
O6—H6B···O3^vi^	0.85	2.00	2.8321 (14)	168

Symmetry codes: (iii) *x*, *y*−1, *z*; (iv) −*x*+1, *y*−1/2, −*z*+1/2; (v) *x*, −*y*+5/2, *z*+1/2; (vi) *x*, *y*+1, *z*.
